# O01 Advancing antimicrobial stewardship through data innovation: a virtual registry for newly licensed antimicrobial agents in Scotland (UKAR: Virtual)

**DOI:** 10.1093/jacamr/dlag102.001

**Published:** 2026-06-26

**Authors:** Cosmika Goswami, Ebru Turgal, Tanja Mueller, Marion Bennie, Rebecca Parr, Gareth T Jones, R Andrew Seaton, David Jenkins, Ioannis Baltas, Jacqueline Sneddon, Jonathan A T Sandoe, Frances Garraghan, Nicholas M Brown, Gary J Macfarlane, Amanj Kurdi

**Affiliations:** Strathclyde University of Pharmacy and Biomedical Sciences, University of Strathclyde, UK; Strathclyde University of Pharmacy and Biomedical Sciences, University of Strathclyde, UK; Strathclyde University of Pharmacy and Biomedical Sciences, University of Strathclyde, UK; Strathclyde University of Pharmacy and Biomedical Sciences, University of Strathclyde, UK; Epidemiology Group, School of Medicine, Medical Sciences and Nutrition, University of Aberdeen, Aberdeen, UK; Epidemiology Group, School of Medicine, Medical Sciences and Nutrition, University of Aberdeen, Aberdeen, UK; British Society for Antimicrobial Chemotherapy, Birmingham, UK; Infectious diseases unit, Queen Elizabeth University Hospital, NHS Greater Glasgow and Clyde, Glasgow, UK; Clinical Microbiology, University Hospitals of Leicester NHS Trust, Leicester UK; British Society for Antimicrobial Chemotherapy, Birmingham, UK; Department of Infection, Immunity and Inflammation, Institute of Child Health, University College London, London, UK; Department of Microbiology, University College Hospital, University College London Hospitals NHS Foundation Trust, London, UK; British Society for Antimicrobial Chemotherapy, Birmingham, UK; Healthcare Associated Infection Group, Leeds Institute of Medical Research, University of Leeds and Leeds Teaching Hospitals NHS Trust, Leeds, UK; British Society for Antimicrobial Chemotherapy, Birmingham, UK; Cambridge University Hospitals NHS Foundation Trust, Cambridge, UK; Epidemiology Group, School of Medicine, Medical Sciences and Nutrition, University of Aberdeen, Aberdeen, UK; Strathclyde University of Pharmacy and Biomedical Sciences, University of Strathclyde, UK; College of Pharmacy, Al-Kitab University, Kirkuk 36015, Iraq; Department of Public Health Pharmacy and Management, School of Pharmacy, Sefako Makgatho Health Sciences University, Pretoria, South Africa; College of Pharmacy, Hawler Medical University, Erbil, Kurdistan region, Iraq

## Abstract

**Background:**

Antimicrobial resistance (AMR) continues to threaten effective infection management, increasing reliance on recently licensed antimicrobials (RLAs) for MDR infections. While these agents are supported by clinical trial data, evidence on their real-world use, effectiveness and safety remains limited. Traditional surveillance systems and clinical registries are resource-intensive and often lack scalability, highlighting the need for innovative, data-driven approaches to support antimicrobial stewardship and policy decision-making.

**Objectives:**

To evaluate the feasibility of using routinely collected electronic healthcare data to establish a national virtual registry capable of monitoring the real-world utilization, microbiology and outcomes of RLAs in Scotland.

**Methods:**

The UK Antimicrobial Registry:Virtual (UKAR:V) Scotland was developed using deterministically linked national datasets. Adult inpatients (≥18 years) prescribed at least one of 11 RLAs (cefiderocol, ceftazidime/avibactam, ceftolozane/tazobactam, meropenem/vaborbactam, imipenem/cilastatin/relebactam, eravacycline, ceftaroline, ceftobiprole, dalbavancin, delafloxacin, oritavancin) between June 2019 and June 2023 were included. Prescribing and administration data were obtained from the Hospital Electronic Prescribing and Medicines Administration (HEPMA) system and linked to national datasets capturing microbiology, hospital admissions, intensive care activity and mortality. Descriptive analyses summarized patient characteristics, prescribing patterns, infection types, microbiological findings and outcomes. Feasibility outcomes included data completeness, linkage success and availability of microbiology and outcome data.

**Results:**

A total of 308 patients received 353 administered RLA prescriptions. Dalbavancin accounted for the majority of use (70.5%), followed by ceftazidime/avibactam (13.3%), ceftolozane/tazobactam (7.9%) and cefiderocol (5.7%). Gram-negative RLAs were predominantly prescribed for severe infections, including respiratory disease and sepsis, and were frequently initiated in intensive care settings. Dalbavancin was commonly used for skin, soft-tissue and device-related infections, often to facilitate early discharge or outpatient management. Microbiology results were available for 35% of patients; *Pseudomonas aeruginosa* and *Klebsiella pneumoniae* were the most frequent Gram-negative isolates, while *Staphylococcus aureus* predominated among Gram-positive infections. Median treatment duration ranged from 7–12 days for Gram-negative RLAs and a single dose for dalbavancin. Twenty-eight day readmission occurred in 25–40% of Gram-negative RLA recipients and 29.8% of dalbavancin recipients, with relapse rates reflecting underlying clinical complexity rather than comparative drug performance. No major data-linkage failures were identified.

**Conclusions:**

UKAR:V demonstrates that routinely collected electronic healthcare data can support scalable, low-burden surveillance of RLAs at a national level. The platform captures clinically meaningful patterns of use and outcomes, complementing traditional registries and supporting antimicrobial stewardship. With further expansion of data coverage and integration of enhanced microbiology and resistance information, UKAR:V provides a sustainable foundation for post-marketing evaluation of RLAs and assessment of innovative reimbursement models, such as the UK subscription scheme.

